# MiR-362-5p, Which Is Regulated by Long Non-Coding RNA MBNL1-AS1, Promotes the Cell Proliferation and Tumor Growth of Bladder Cancer by Targeting QKI

**DOI:** 10.3389/fphar.2020.00164

**Published:** 2020-03-03

**Authors:** Xiaosong Wei, Beibei Wang, Qi Wang, Xiaoming Yang, Yang Yang, Zhiwei Fang, Chengzhi Yi, Lei Shi, Xin Fan, Jin Tao, Yufeng Guo, Dongkui Song

**Affiliations:** ^1^ Department of Urology, The First Affiliated Hospital of Zhengzhou University, Zhengzhou, China; ^2^ Department of Pathology, The First Affiliated Hospital of Zhengzhou University, Zhengzhou, China; ^3^ College of Science, The Australian National University, Canberra, ACT, Australia

**Keywords:** MiR-362-5p, bladder cancer, QKI, lncRNA MBNL1-AS1, proliferation

## Abstract

In this study, we found miR-362-5p was upregulated in bladder cancer tissues and we predicted that QKI is potential a target of miR-362-5p and MBNL1-AS1 might be able to directly target to miR-362-5p. We attempted to evaluate whether miR-362-5p could play its roles in bladder cancer through regulating QKI (quaking) and whether the expression and function of miR-362-5p could be mediated by lncRNA MBNL1-AS1. We performed the gain- and loss-function experiments to explore the association between miR-362-5p expression and bladder cancer proliferation. *In vivo*, the nude mice were injected with miR-362-5p knockdown SW780 cells to assess the effects of miR-362-5p on tumor growth. The results showed upregulation of miR-362-5p promoted cell proliferation of bladder cancer cells. MBNL1-AS1 and QKI could directly bind with miR-362-5p, and knockdown of MBNL1-AS1 or QKI could abrogate the regulatory effects of miR-362-5p on bladder cancer cell proliferation. Furthermore, downregulation of miR-362-5p inhibited bladder tumor growth and increased QKI expression. Our data unveiled that miR-362-5p may play an oncogenic role in bladder cancer through QKI and MBNL1-AS1 might function as a sponge to mediate the miR-362-5p expression and function.

## Introduction

Bladder cancer is a common urological malignancy worldwide in the urinary system and occurs more frequently in men than women (Ferlay et al., 2015; Antoni et al., 2017). Bladder cancers include low-grade non-muscle-invasive bladder cancers (NMIBCs) and high-grade muscle-invasive bladder cancers (MIBCs), and >90% of all bladder cancer is urothelial carcinoma (Jemal et al., 2010; Verdoorn et al., 2013). Currently, therapeutic methods are transurethral resection of the bladder tumor for NMIBCs and radical cystectomy for MIBCs. However, the bladder cancer has a high rate of recurrence. The 5–30% of these NMIBCs cases will develop to MIBCs and MIBCs have a poor 5-year overall survival (approximately 15–70%) (Shabsigh et al., 2009; Prasad et al., 2011; Knowles and Hurst, 2015). Therefore, a better understanding of the molecular mechanisms underlying carcinogenesis and progression is needed for bladder cancer.

MicroRNAs (miRNAs) are a class of small non-coding RNAs (about 22 nucleotides in length) and negatively regulate gene expression at the post-transcriptional level by binding to specific complementary messenger RNAs (Bartel, 2004; Croce and Calin, 2005; Bartel, 2009). Growing evidences have proved that miRNAs function as oncogenes or tumor suppressors by mediating the cancer cell proliferation, apoptosis, invasion, tumor angiogenesis, and metastasis (Zhang et al., 2007; Fendler et al., 2011; Van Roosbroeck and Calin, 2017). Numbers of miRNAs play their roles in various kinds of cancers including bladder cancer. For example, miR-143, miR-490-5p, and miR-26a have been regarded as tumor suppressors by inhibiting the proliferation of bladder cancer (Lin et al., 2009; Lin et al., 2013; Lan et al., 2015). Oppositely, knockdown of miR-221 suppresses cell apoptosis in human bladder cancer and miR-182-5p promotes the proliferation of bladder cancer cell (Lu et al., 2010; Xie et al., 2018).

The biological functions of miR-362-5p have been extensively reported in various types of cancers. Increasing numbers of studies indicate that miR-362-5p promotes cell proliferation, tumor growth, and metastasis in hepatocellular carcinoma, human breast cancer, and lung cancer (Ni et al., 2015; Ni et al., 2016; Luo et al., 2018). However, miR-362-5p inhibits proliferation and migration of neuroblastoma cells and it is downregulated in renal cell carcinoma (Wu et al., 2015; Ying et al., 2018). It indicates that miR-362-5p could play oncogenic or suppressive roles in progression of these cancers. In the present study, we found that the expression of miR-362-5p was significantly upregulated in bladder cancer tissues compared to adjacent tissues. However, the biological function of miR-362-5p in bladder cancer has not been studied. Therefore, we tried to reveal the roles of miR-362-5p in bladder cancer and its underlying mechanism.

Based on biometric analysis (Starbase, http:starbase.sysu.edu.cn), RNA binding protein QKI (quaking) is one of the potential bind targets of miR-362-5p. Importantly, the expression of QKI is downregulated in bladder cancer tissues (Novikov et al., 2011). QKI is the number of signal transduction and activator of RNA (STAR) family and has three major isoforms (QKI5, -6, and -7) (Larocque and Richard, 2005). QKI has been demonstrated in the regulation of cellular processes such as cell cycle, apoptosis, and differentiation (Guo et al., 2011; Yang et al., 2011; Shu et al., 2017). Studies found that QKI could inhibit the proliferation and tumorigenesis in multiple cancer types such as colon cancer, gastric cancer, lung adenocarcinoma, and renal cell carcinoma (Yang et al., 2010; Bian et al., 2012; Zhang et al., 2016; Zhang et al., 2019). Therefore, in the present study, we want to study whether miR-362-5p could play its roles through regulating QKI.

Long non-coding RNAs (lncRNAs, > 200 nt) are another class of ncRNAs, which have been proposedas competing endogenous RNAs (ceRNAs) or “RNA sponges” to sequester the miRNAs, leading to attenuate miRNAs' regulatory effects on target mRNAs (Ebert et al., 2007; Cesana et al., 2011). In this study, we predicted and verified that miR-362-5p directly binds with lncRNA MBNL1-AS1 and the expression of MBNL1-AS1 is significantly decreased in Bladder Urothelial Carcinoma tissues (GEPIA, http://gepia.cancer-pku.cn/). Therefore, we hypothesized that functions miR-362-5p/QKI axis in bladder cancer could be mediated by MBNL1-AS1. To verify our hypothesis, we tried to assess the effects of miR-362-5p on the proliferation of bladder cancer cells *in vitro* and the tumor growth *in vivo*, and study whether the functions of miR-362-5p are related to MBNL1-AS1 and QKI in bladder cancer.

## Materials and Methods

### Clinical Samples

This study was approved by the Ethics Committee of The First Affiliated Hospital of Zhengzhou University (Zhengzhou, China). All procedures were in accordance with the Declaration of Helsinki. Written informed consents were obtained from all the patients with bladder cancer who were histopathologically and clinically diagnosed. A total of 24 pairs of bladder cancer tissues and matched adjacent tissues were collected from the patients who did not undergo radiotherapy or chemotherapy before surgery. The adjacent tissues were normal bladder tissues that collected at more than 3 cm away from the tumor tissues (Li et al., 2019a). All the tissues were immediately frozen in liquid nitrogen after surgical operation and stored at −80°C till use.

### Cell Culture

The human bladder carcinoma cell lines 5637, SW780, and UMUC3 cell lines were purchased from Procell (Wuhan, China) and T-24 cells was purchased from zhongqiaoxinzhou (Shanghai, China). All cell lines were grown in appropriate medium: 5637 and T-24 cells were cultured in RPMI1640 medium (31800-022; Gibco, Grand Island, NY, USA), SW780 cells were cultured in DMEM (12100-046; Gibco), and UMUC3 cells were cultured in MEM (41500-067; Gibco), supplemented with 10% fetal bovine serum (FBS) (SH30084.03; Hyclone, South Logan, UT, USA) at 37°C in a humidified atmosphere with 5% CO_2_.

### Cell Transfection and Infection

Two short hairpin RNAs (shRNA, shRNA-2) against MBNL1-AS1 or a negative control shRNA (NC shRNA) were inserted into PRNAH1.1 vector and purchased from GenScript (Nanjing, China). SiRNA against QKI (Yang et al., 2011), NC siRNA, miR-362-5p mimic, NC mimic, miR-362-5p inhibitor, and NC miRNA inhibitor were synthesized and purchased from JTS (Wuhan, China). The sequences were listed in **Table 1**.

**Table 1:** The RNA sequences used in this study.

**Gene name**		**Sequence (5′–3′)**
MBNL1-AS1 shRNA	Sense	GATCCGAACGAAAGGAGCAGGGTATTTCAA GAGAATACCCTGCTCCT TTCGTTTTTTTA
	Antisense	AGCTTAAAAAAACGAAAGGAGCAGGGTAT TCTCTTGAAATACCCTGCTCCTTTCGTTCG
MBNL1-AS1 shRNA-2	Sense	GATCCGCCAGAACCTAGTCTCATGTTTCAA GAGAACATGAGACTAGGTTCTGGTTTTTA
	Antisense	AGCTTAAAAACCAGAACCTAGTCTCATGTT CTCTTGAAACATGAGACTAGGTTCTGGCG
NC shRNA	Sense	GATCCCCTTCTCCGAACGTGTCACGTTTCA AGAGAACGTGACACGTTCGGAGAATTTTT
	Antisense	AGCTAAAAATTCTCCGAACGTGTCACGTTC TCTTGAAACGTGACACGTTCGGAGAAGGG
QKI siRNA	Sense	CCUUGAGUAUCCUAUUGAACCUAGU
	Antisense	ACUAGGUUCAAUAGGAUACUCAAGG
NC siRNA/NC mimic	Sense	UUCUCCGAACGUGUCACGUTT
	Antisense	ACGUGACACGUUCGGAGAATT
miR-362-5p mimic	Sense	AAUCCUUGGAACCUAGGUGUGAGU
	Antisense	UCACACCUAGGUUCCAAGGAUUUU
Anti-miR-362-5p	Sense	GATCCACTCACACCTAGGTTCCAAGGATTTT CAAGAGAAATCCTTGGAACCTAGGTGTGAG TTTTTTTA
	Antisense	AGCTTAAAAAAACTCACACCTAGGTTCCAAG GATTTCTCTTGAAAATCCTTGGAACCTAGGT GTGAGTG
Anti-miRNA-NC	Sense	GATCCTTCTCCGAACGTGTCACGTTTCAA GAGAACGTGACACGTTCGGAGAATTTTTTA
	Antisense	AGCTTAAAAAATTCTCCGAACGTGTCACGT TCTCTTGAAACGTGACACGTTCGGAGAAG
miR-362-5p inhibitor		ACUCACACCUAGGUUCCAAGGAUU
NC inhibitor		UUGUACUACACAAAAGUACUG

Cells (4×10^5^) were seeded into six-well plates and performed cell transfection in using Lipofectamine 2000 reagent (11668-019; Invitrogen, Carlsbad, MA, USA) according to the manufacturer's instructions. The cells were transfected with 100 pmol of miR-362-5p mimic, miR-362-5p inhibitor, QKI siRNA, or 2 μg of MBNL1-AS1 shRNA. The 5637 cells were co-transfected with 1 μg of MBNL1-AS1 shRNA with 50 pmol of miR-362-5p inhibitor. The SW780 cells were co-transfected with 50 pmol of miR-362-5p inhibitor and 50 pmol of QKI siRNA. After 4 h transfection, the cells were cultured in complete medium and used for subsequent experiments.

The adenoviral vector pAdTrack-CMV (Addgene #16405) expressing MBNL1-AS1 (NR 027038.1) (Ad-MBNL1-AS1) were constructed and purchased from GenScript. The SW780 cells were infected with Ad-MBNL1-AS1 or Ad-NC. After 24 infection, the cells were cultured in complete medium and used for subsequent experiments.

### Immunofluorescent Assay

5-Bromo-2-deoxyuridine (BrdU) incorporation into DNA of proliferating cells was used to measure cell proliferation. In this study, immunofluorescent assay was used to determine the BrdU positive cells. Briefly, after transfection 48 h, the cells were added 10 µM BrdU for 24 h for stabilization. Then cells were fixed in a solution containing 4% paraformaldehyde (80096618; Sinopharm, Shanghai, China). After incubating in 0.1% Triton X-100 (ST795; Beyotime, Shanghai, China) and blocking with goat serum (SL038; Solarbio, Beijing, China), the cells were incubated with BrdU antibody (66241-1; Proteintech, Wuhan, China) at 4 °C overnight. After washing, the cells were incubated with secondary antibody at room temperature for 1 h and stained with DAPI (C1002; Beyotime). Cells were then observed using a microscope (400×, IX53; Olympus, TKY, Japan) and photographed (DP73; Olympus). The percentage of BrdU positive cells was measured.

### MTT Assay

Cell proliferation and viability was assessed using 3-(4,5-dimethylthiazol-2-yl)-2,5-diphenyl tetrazolium (MTT, M-2128; Sigma-Aldrich, St. Louis, MO, USA)-based assay. In brief, the transfected cells (4×10^3^) were seeded in 96-well plates and cultured in culture medium at 37 °C for 0, 24, 48, 72, and 96 h, respectively. For cell viability determination, the cells were cultured for 48 h before MTT treatment. After each indicated timepoint, the cells were changed into fresh medium containing 0.5mg/ml MTT and cultured at 37 °C for 4 h. After removing the culture medium, 150 μl DMSO was added to each well to dissolve the crystals. The absorption values were measured at 570 nm on a multi-well plate reader after 10 min incubation in darkness.

### Quantitative Real-Time PCR (qRT-PCR)

The total RNA was extracted with the RNAsimple Total RNA Kit (DP419; Tiangen, Beijing, China) according to the manufacturer's instructions. For miR-362-5p and control U6 detection, the cDNA was generated by reverse-transcription system including primers, dNTP, Buffer, RNase inhibitor (DP418; Tiangen), ddH_2_O, and reverse transcriptase TIANSeq M-MLV (NG212; Tiangen) in a 20 μl reaction. The mixture was incubated at 37 °C for 30 min and then incubated 42 ° for 30 min. After stopping the reaction at 70 °C for 10 min. The cDNA was used for qRT-PCR detection. The reverse-transcription primers for miR-362-5p and U6 were 5′-GTTGGCTCTGGTGCAGGGTCCGAGGTATTCGCACCAGAGCCAACACTCAC-3′ and 5′-GTTGGCTCTGGTGCAGGGTCCGAGGTATTCGCACCAGAGCCAACAAAATATGG-3′, respectively. For lncRNA MBNL1-AS1 and gene mRNA detection, the mixture including oligo (dT), random primer, and ddH_2_O in total 12.5 μl was incubated at 70 °C for 5 min, then put it on ice for 2 min and then centrifuged briefly. The reaction supernatant was added into the mixture containing dNTP, Buffer, Rnase inhibitor, and reverse transcriptase TIANSeq M-MLV in a 20 μl reaction. The mixture was incubated at 25 °C for 10 min and then incubated 42 °C for 50 min. After stopping the reaction at 80 °C for 10 min. The cDNA was used for qRT-PCR detection. The qRT-PCR was carried out by using 2× Taq PCR MasterMix (KT201; Tiangen) and SYBR Green (SY1020; Solarbio) and on the Exicycler™ 96 detection system (BIONEER, Daejeon, Korea). The primer sequences (GenScript, Nanjing, China) of qRT-PCR used in the study were listed in **Table 2**. The relative expression of targets was calculated using the 2^−ΔΔCt^ method. Expression of small non-coding RNA U6 was used as endogenous control of miR-362-5p and GAPDH was used as endogenous control of MBNL1-AS1, QKI, and cylindromatosis (CYLD, a well-known target of miR-362-5p).

**Table 2:** The primer sequences of qRT-PCR used in this study.

**Primer name**		**Sequence (5′–3′)**
hsa-miR-362-5p	Forward	CCTTGGAACCTAGGTGTGAGTG
	Reverse	TGGTGCAGGGTCCGAGG
U6	Forward	GCTTCGGCAGCACATATACT
	Reverse	GTGCAGGGTCCGAGGTATTC
MBNL1-AS1	Forward	TGGATAAGACAGTCCCTACA
	Reverse	ATTGGATTGCTTCCCACATA
QKI	Forward	ACGGAACTCCTCACCCA
	Reverse	CCGCACCTAATACACCACT
CYLD	Forward	TAATAAACCAAAGGCTACAGG
	Reverse	TGGTGAAGAACGGTCAAAGT
GAPDH	Forward	GACCTGACCTGCCGTCTAG
	Reverse	AGGAGTGGGTGTCGCTGT

### Cell Cycle Analysis

Cell cycle distribution was analyzed using a flow cytometer (NovoCyte; Aceabio, San Diego, CA, USA). Cells (4×10^5^/well) were plated in six-well plates. The transfection experiments were performed. After incubation for 48 h, cells were collected and fixed with 70% chilled ethanol. After washing, cells were incubated with nuclei-staining buffer containing PI/RNase A (C1052;Beyotime) for 30 min in dark according to the protocol and kept on ice before analysis.

### Luciferase Activity Assay

The reconstructed pmirGLO luciferase vectors (GenScript) contained the fragment of 3′UTR of wild-type QKI (QKI-Wt) or fragment of MBNL1-AS1 (MBNL1-AS1-Wt) with miR-362-5p binding sites or without miR-362-5p binding sites (mutant QKI (QKI-Mut) and MBNL1-AS1 (MBNL1-AS1-Mut)). For luciferase activity measurement, 293T cells were seeded into plates and co-transfected with the reconstructed luciferase vector and miR-362-5p mimic/NC mimic by Lipofectamine 2000 reagent. After 48 h transfection, luciferase activity was detected by the Dual Luciferase Reporter Gene Assay Kit (KGAF040; KeyGen Biotech, China) according to the manufacturer's instructions. The results were expressed as the ratio of Renilla luciferase activity to firefly luciferase activity.

### Western Blot

Total protein was extracted from tissues and cells using RIPA lysis buffer (R0010; Solarbio) and protein concentration was quantified using BCA protein assay kit (PC0020; Solarbio). Twenty micrograms of total protein from each sample was subjected to SDS-PAGE using 10% polyacrylamide gels and transferred to PVDF membranes (IPVH00010; Millipore, Billerica, MA, USA). The membranes were blocked with 5% non-fat milk and then incubated with primary antibodies against CYLD (1:1,000; 11110-1-AP, Proteintech), QKI (1:1,000; 13169-1-AP, Proteintech), poly (ADP-ribose) polymerase 1 (PARP-1) (1:1,000; 22999-1-AP, Proteintech), p27 (1:2,000; 25614-1-AP, Proteintech), E2F1 (1:1,000; 12171-1-AP, Proteintech), macroH2A1.1 (1:1,000; A7045, ABclonal, Wuhan, China), cyclin D (1:1,000; A0310, ABclonal), c-Fos (1:1,000; A0236, ABclonal), macroH2A1.2 (1:1,000; #4827, CST, Danvers, MA, USA), and GAPDH (1:10,000; 60004-1, Proteintech) at 4°C overnight. After washing, the membranes were incubated with horseradish peroxidase (HRP)-conjugated sheep anti-rabbit IgG or anti-mouse IgG antibody for 1 h at 37°C. The enhanced chemiluminescence kit (ECL, PE0010; Solarbio) was used to detect the signals of protein bands and normalized to GAPDH.

### Immunohistochemical Analysis

Immunohistochemical detection of Ki-67 in paraffin embedded tumor tissue sections were processed for classical immunohistochemical procedures. Non-specific binding was blocked by incubating sections with goat serum for 15 min at room temperature, followed by incubation at 4°C overnight with anti-Ki-67 antibody (1:500, ab15580; Abcam, Cambridge, UK). The sections were washed with PBS three times and stained with HRP-conjugated goat anti-rabbit IgG antibody (1:500; #31460, Thermo Fisher Scientific, Waltham, MA, USA) at 37°C for 1 h. The sections were treated with diaminobenzidine substrate (DAB, DA1010; Solarbio) and counterstained with hematoxylin. The staining results of Ki-67 in tumor tissues were observed using a microscope (400×, BX53; Olympus) and photographed (DP73; Olympus).

### Animal Experiments

In order to knockdown miR-362-5p expression, the pRNA-H1.1/Adeno vector inserted with a hairpin sequence containing the 100% complementary nucleotide sequence of miR-362-5p (termed anti-miR-362-5p) (Baraniskin et al., 2012). The constructed pRNA-H1.1/Adeno vectors containing anti-miR-362-5p or a scramble sequence (NC) were constructed and purchased Wanleibio (Shenyang, China). The hairpin sequence of anti-miR-362-5 and NC listed in **Table 1**.

For cell stable transfection, SW780 cells were transfected with pRNA-H1.1/Adeno vectors contained with anti-miR-362-5p or NC and then selected using 300 mg/ml G418 (11811023; Invitrogen). After protein and miR-362-5p expression detection, the cells were ready for xenograft mouse model assay. Balb/c nude mice (6 weeks) were purchased from the HFK Bioscience (Beijing, China). The mice were maintained under specific pathogen-free conditions in an environmentally controlled room with a 12-h light/12-h dark cycle and a constant temperature of 22 ± 1°C. The mice were provided with a standard diet and water. For investigating the role of miR-362-5p in bladder tumor growth, 12 mice were randomly divided into two groups (anti-miR-362-5p group and NC group). The mice were injected subcutaneously into the left flank with 1×10^7^ SW780 cells/100 μl PBS that were transfected with anti-miR-362-5p or NC. The tumor diameters were measured every 3 days from day 7 to day 25 with calipers, and the tumor volumes were calculated according to the following formula: [(width)^2^ × length]/2. The mice were sacrificed on day 25 and the tumors were dissected and weighed. The study was conducted in strict compliance with the recommendations set forth in the Guideline for the care and use of laboratory animals. The animal protocols were approved by the Ethics Committee of The First Affiliated Hospital of Zhengzhou University.

### Statistical Analysis

All data were analyzed using GraphPad Prism^®^ 7 software and expressed as mean ± Standard Deviation (SD). Data between the control group and the experimental group were analyzed by Student's t-tests, and data with multiple groups were analyzed by analysis of variance (ANOVA). The Spearman correlation coefficient analysis was evaluated the correlations between the levels of miR-362-5p and MBNL1-AS1 verified by qRT-PCR. The data of clinical specimens were analyzed using paired t-test. P < 0.05 was considered statistically significant.

## Results

### Expression of MiR-362-5p in Clinical Bladder Cancer Tissue

**Figure 1:**
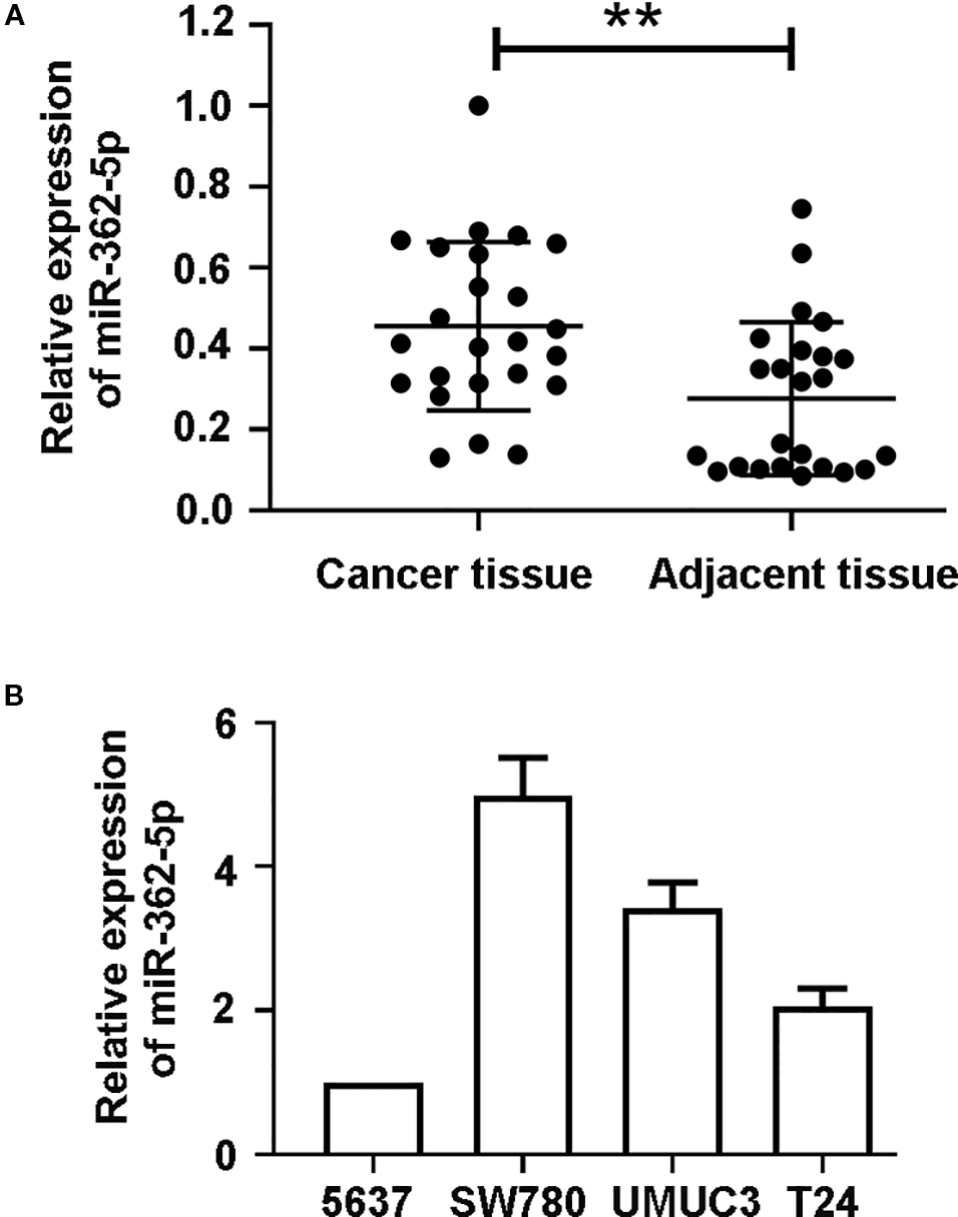
MiR-362-5p is upregulated in bladder cancer tissues. **(A)** The relative expression levels of miR-362-5p in bladder cancer tissues (N = 24) and adjacent tissues (N = 24) were analyzed by qRT-PCR. **p < 0.01 *vs*. adjacent tissues. **(B)** The expression levels of miR-362-5p in bladder cancer cell lines 5637, SW780, UMUC3, and T24 were analyzed by qRT-PCR.

The 24 pairs of clinical bladder cancer tissues and adjacent tissues without radiotherapy and chemotherapy were collected and the expression of miR-362-5p was detected by qRT-PCR. The expression of miR-362-5p was significantly higher than that in adjacent tissues (**Figure 1A**). We chose four bladder cancer cell lines to detect miR-362-5p expression. According to the results, we selected 5637 cell (miR-362-5p lower expression) and SW780 cell (miR-362-5p higher expression) for the further study (**Figure 1B**).

### MiR-362-5p Promotes the Proliferation of Bladder Cancer Cells

**Figure 2:**
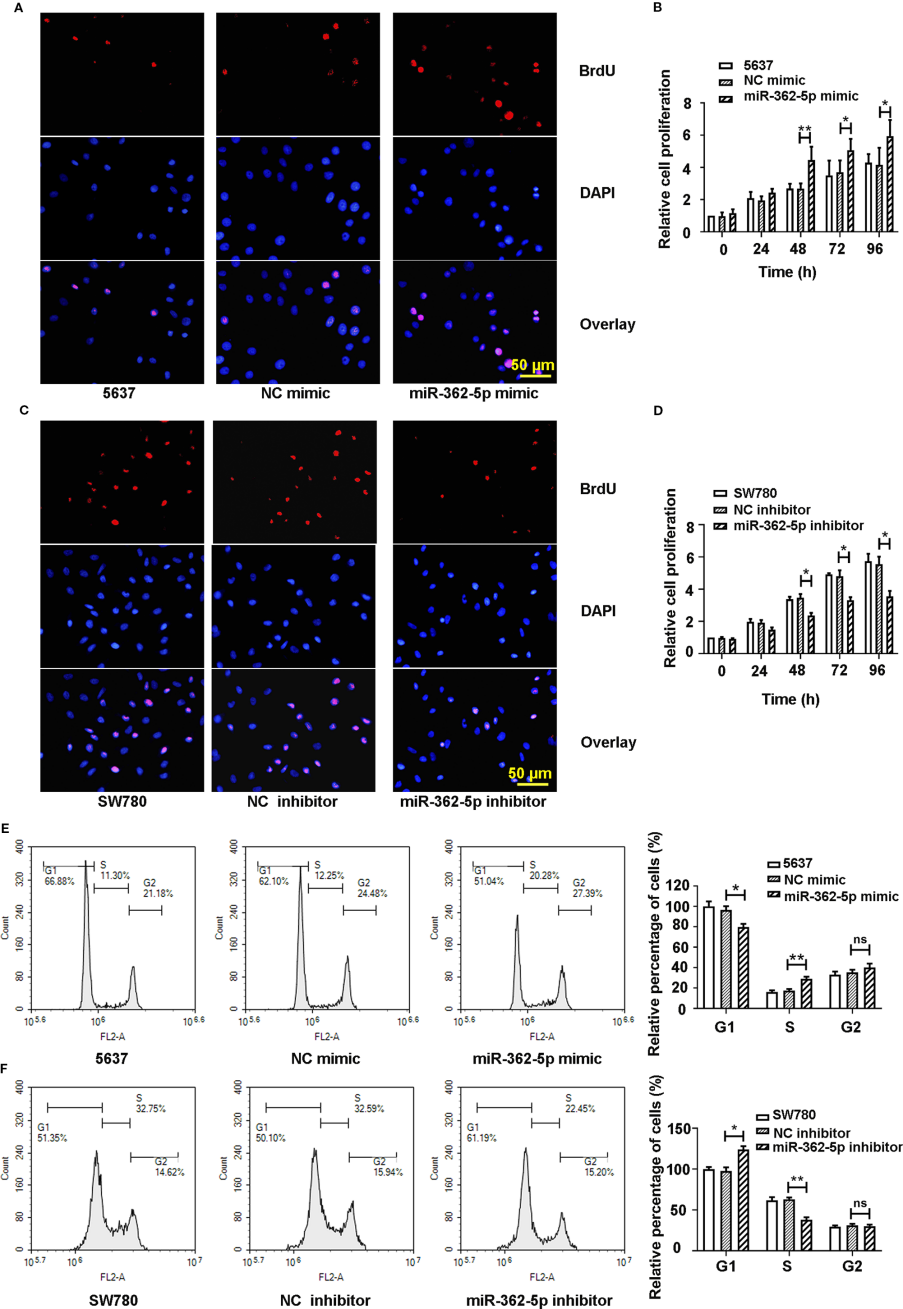
The miR-362-5p promotes the cell proliferation of bladder cancer. **(A, C)** The 5637 cells were transfected with miR-362-5p mimic (100 pmol) or NC mimic; the SW780 cells were transfected with miR-362-5p inhibitor (100 pmol) or NC inhibitor. After 48 h, the cell proliferation was examined by staining with BrdU in immunofluorescence assay (bar=50 μm). **(B, D)** Cell proliferation of miR-362-5p mimic/NC mimic tranfected 5637 cells and miR-362-5p inhibitor/NC inhibitor transfected SW780 cells was also measured by MTT assay. The cell proliferation was displayed as fold of 5637 or SW780 cells at 0 h. **(E, F)** The cell cycle distribution of miR-362-5p mimic/NC mimic tranfected 5637 cells and miR-362-5p inhibitor/NC inhibitor transfected SW780 cells was measured using flow cytometer. The percentage of cells was displayed as fold of 5637 or SW780 cells in G1 phase. **p < 0.01 and *p < 0.05 *vs*. corresponding controls.

To investigate the effect of miR-362-5p on the proliferation of bladder cancer cells, the 5637 cells were transfected with miR-362-5p mimic or NC mimic, and SW780 cells were transfected with miR-362-5p inhibitor or NC inhibitor. We determined the efficiency of gain- and loss of miR-362-5p by detecting the expression of miR-362-5p in four bladder cancer cell lines (**Supplementary Figure 1A, B**). Additionally, we also determined the transfection efficiency in 5637 and SW780 cells by detecting the expression of CYLD, a well-known target for miR-362-5p (Ni et al., 2015) (**Supplementary Figures 1C, D, F, G**). Cell proliferation and cycle distribution were measured after 48 h transfection. Upregulation of miR-362-5p increased BrdU positive cells and cell proliferation of 5637 cells since 48 h compared to NC mimic transfected cells (**Figures 2A, B**, **Supplementary Figure 1I**). Conversely, downregulation of miR-362-5p remarkably inhibited the proliferation of SW780 cells (**Figures 2C, D**, **Supplementary Figure 1I**). The results show that upregulation of miR-362-5p could promote G1/S transition in the progression of cell cycle, whereas downregulation of miR-362-5p arrested the cell at G1 phase (**Figures 2E, F**). We also verified our findings using miR-362-5p/NC mimic transfected T-24 cell and miR-362-5p/NC inhibitor transfected UMUC3 cells by MTT assay. And we got the similar results (**Supplementary Figures 1J, K**). The results indicated that miR-362-5p promotes the proliferation of bladder cancer cells.

### MiR-362-5p are Directly Binding With QKI

**Figure 3:**
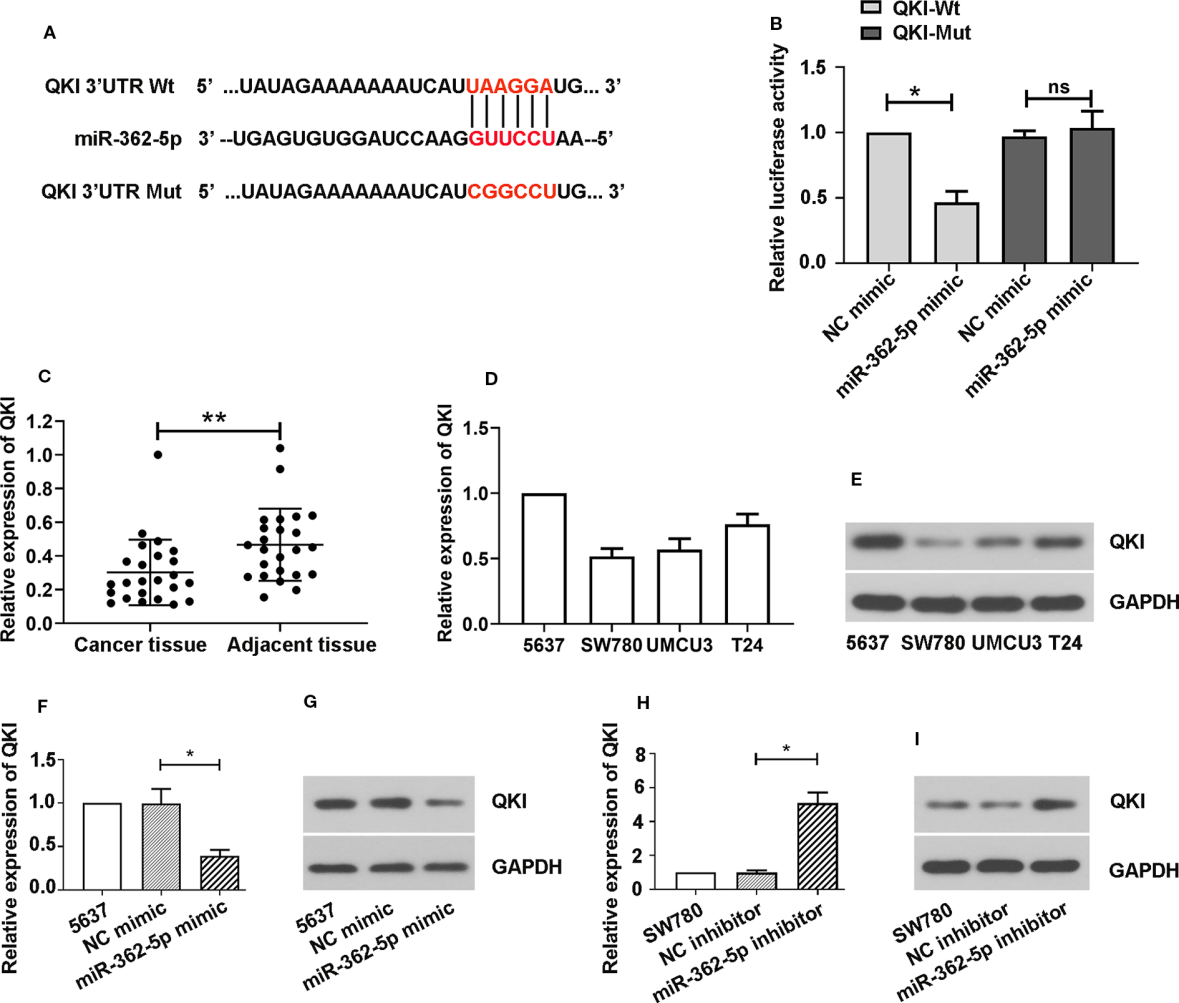
MiR-362-5p directly targets with QKI. **(A)** The predicted binding sites within miR-362-5p and wild-type of 3′UTR of QKI (QKI-Wt) and the mutant sequence of 3′UTR of QKI (QKI-Mut). **(B)** The pmirGLO luciferase vector was inserted with fragments of QKI-Wt or QKI-Mut. Then the constructed pmirGLO luciferase vectors (1 μg) were co-transfected with miR-362-5p mimic (50 pmol) or NC mimic. Luciferase reporter assay was performed after for 48 h transfection. *p < 0.05 *vs*. NC controls. **(C)** The relative expression levels of QKI in bladder cancer tissues (N=24) and adjacent tissues (N=24) were analyzed by qRT-PCR. **p < 0.01 *vs*. adjacent tissues. **(D, E)** The mRNA and protein expression levels of QKI in bladder cancer cell lines 5637, SW780, UMUC3, and T24 were analyzed by qRT-PCR. **(F, G)** The 5637 cells were transfected with miR-362-5p mimic (100 pmol) or NC mimic for 48 h. The mRNA and protein levels of QKI were detected by qRT-PCR and western blot. **(H, I)** The SW780 cells were transfected with miR-362-5p inhibitor (100 pmol) or NC inhibitor for 48 h. The mRNA and protein levels of QKI were detected by qRT-PCR and western blot. The expression was displayed as fold of 5637 or SW780 cells. GAPDH was used as an internal control in western blot. *p < 0.05 *vs*. corresponding controls.

We predicted the binding sites of miR-362-5p and 3′UTR of wild type of QKI (QKI-Wt) and sequence the 3′UTR of QKI mutant (QKI-Mut) that were indicated in red (**Figure 3A**). The luciferase vectors containing fragment of 3′UTR of QKI-Wt or QKI-Mut was co-transfected with miR-362-5p mimic or NC mimic into 293T cells. The results showed that miR-362-5p mimic reduced the luciferase activity in QKI-Wt transfected cells but not affected on the QKI-Mut transfected cells, indicating that miR-362-5p directly and specifically binds with QKI (**Figure 3B**). We measured the expression of QKI in bladder cancer tissue and adjacent tissue (**Figure 3C**). And the mRNA and protein levels of QKI were measured in four cancer cell lines (**Figures 3D, E**). The results were showed that QKI expression was decreased in bladder cancer tissue and it was oppositely with miR-362-5p in bladder cancer tissue and cell lines. For further confirmation, we found that miR-362-5p expression could negatively regulate mRNA and protein expression of QKI in bladder cancer cells (**Figures 3F–I**).

### MiR-362-5p Mediates the Proliferation of Bladder Cancer Cells Through QKI

To determine whether miR-362-5p mediates bladder cancer cell proliferation *via* QKI, we co-transfected miR-362-5p inhibitor/NC inhibitor and QKI siRNA/NC siRNA into SW780 cells. We determined the efficiency of siRNA of QKI and the effects of knocking down QKI on bladder cancer cell proliferation. The results showed that QKI siRNA could significantly decrease the mRNA and protein levels of QKI both in SW780 and 5637 cells (**Supplementary Figures 2A–D**). And knockdown of QKI could promote cell proliferation of bladder cancer cells (**Supplementary Figures 2E, F**).

**Figure 4:**
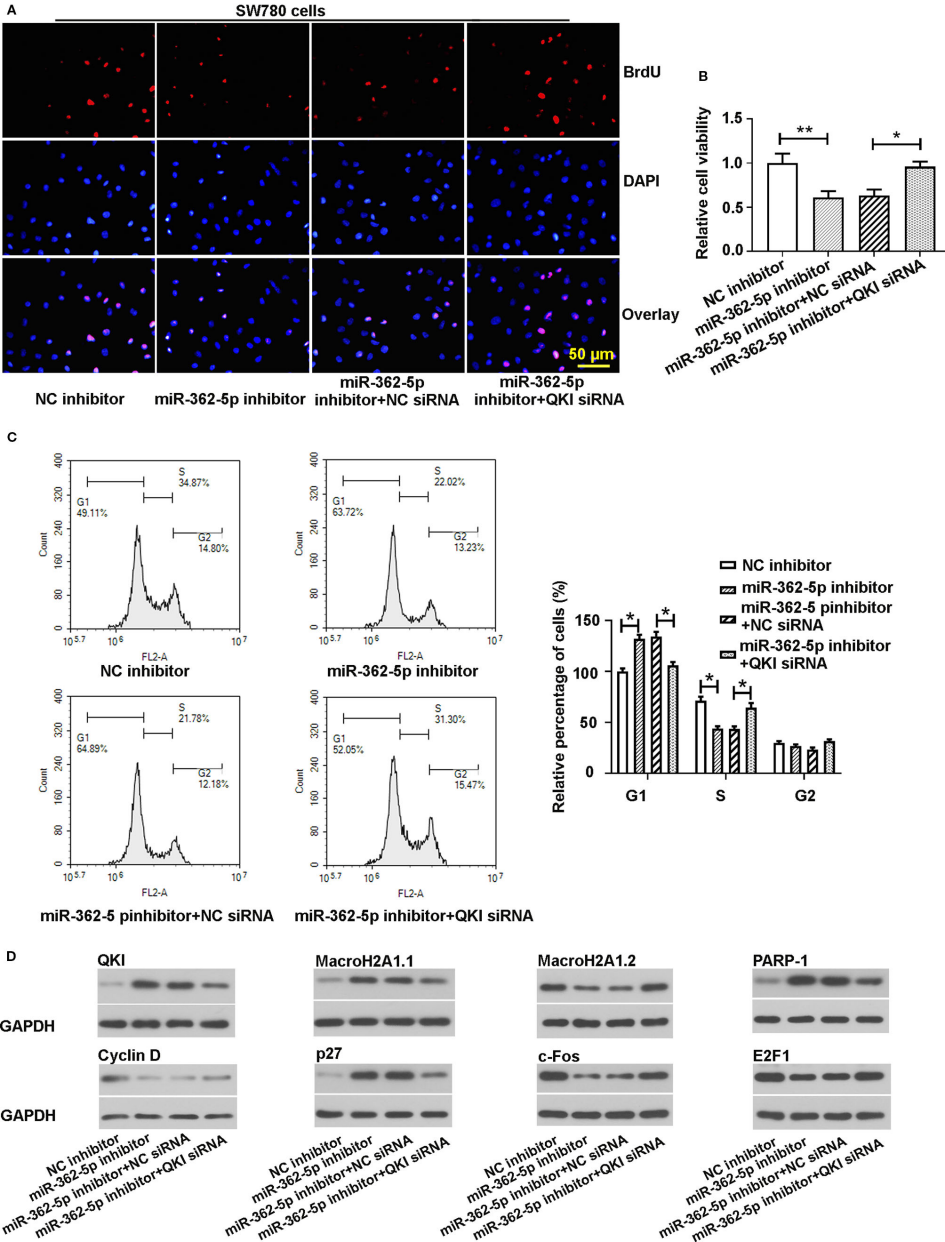
Knockdown of QKI abates the effects of miR-362-5p inhibition on the proliferation of bladder cancer cells. **(A, B)** The SW780 cells were co-transfected with miR-362-5p inhibitor/NC inhibitor (50 pmol) and QKI siRNA/NC siRNA (50 pmol) for 48 h. Then the cell proliferation and cell viability were determined by staining BrdU (bar=50 μm) and MTT assay. The cell viability was displayed as fold of NC inhibitor. **(C)** The cell cycle distribution of the transfected cells was measured using flow cytometer after 48 h transfection. The percentage of cells was displayed as fold of NC inhibitor in G1 phase. **(D)** The protein levels of QKI, Cyclin D, MacroH2A1.1, MacroH2A1.2, p27, c-Fos, PARP-1, and E2F1 in the transfected cells were measured by western blot analysis after 48 h transfection. GAPDH was used as an internal control in western blot. **p < 0.01 and *p < 0.05 *vs*. corresponding controls.

Moreover, downregulation of QKI suppressed the decreased proliferation and cell viability that caused by miR-362-5p inhibitor (**Figures 4A, B**, **Supplementary Figure 2G**). In addition, silencing QKI reduced the cell arrest in the G1 phase that induced by downregulation of miR-362-5p (**Figure 4C**). Western blot analysis was used to measure the expressions of QKI and the cell proliferation-related gene proteins. The results displayed that downregulation of miR-362-5p increased the expression levels of QKI, MacroH2A1.1, PARP-1, and p27. And downregulation of miR-362-5p decreased the protein levels of Cyclin D, MacroH2A1.2, and c-Fos but it did not much affect E2F1 expression (**Figure 4D**). And knocking down QKI could attenuate the protein expression changes that mediated by downregulation of miR-362-5p in SW780 cells.

### Downregulation of MiR-362-5p Inhibits Tumor Growth in Xenograft Mouse Model

**Figure 5:**
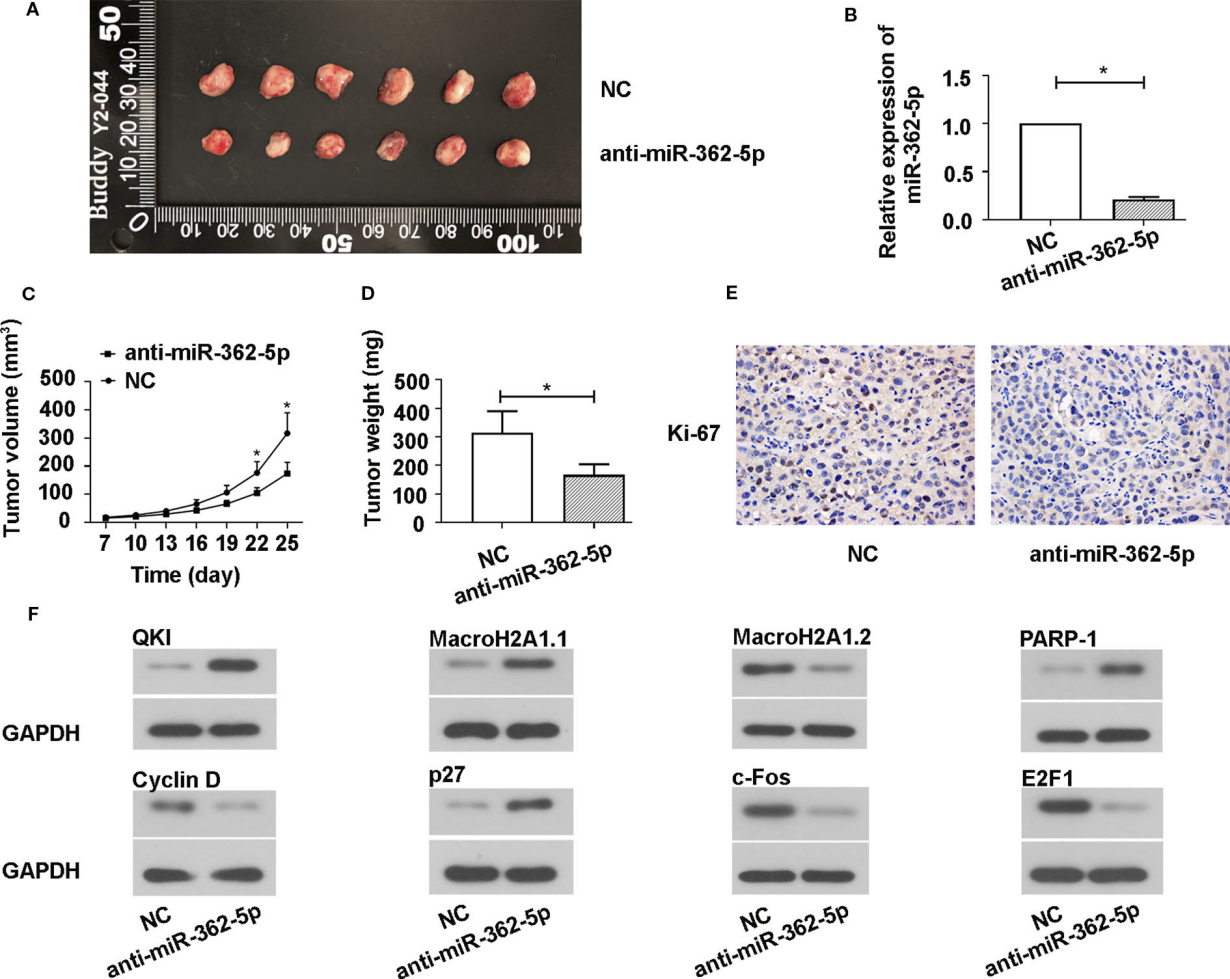
Downregulation of miR-362-5p inhibits the SW780 xenograft tumor growth. **(A)** A hairpin sequence containing the 100% complementary nucleotide sequence of miR-362-5p was constructed into pRNA-H1.1/Adeno vector. The SW780 cells were transfected with vector containing anti-miR-362-5p (2 μg) or miR-NC. Then transfected SW780 cells were selected using 300 µg/ml G418. The Balb/c nude mice were injected subcutaneously into the left flank with 1×10^7^ SW780 cells stable transfected cells (100 μl PBS) to form xenograft tumors. The tumor volumes were measured on day 25. **(B)** The relative expression of miR-362-5p in xenograft tumor tissues from anti-miR-362-5p or miR-NC injected mice were analyzed by qRT-PCR. The expression was displayed as fold of NC. **(C)** The tumor growth curves were determined by assessing the tumor volumes every 3 days from day 7 to day 25 by measuring two perpendicular diameters and the tumor volumes were calculated according to the following formula: [(width)^2^ × length]/2. **(D)** The xenograft tumor weight was measured on day 25. **(E)** The expression levels of Ki-67 in the xenograft tumor tissues were determined using immunohistochemical assay. **(F)** The protein levels of QKI, Cyclin D, MacroH2A1.1, MacroH2A1.2, p27, c-Fos, PARP-1, and E2F1 in xenograft tumor tissues were detected by western blot. GAPDH was used as an internal control in western blot. *p < 0.05 *vs*. NC controls.

To investigate whether miR-362-5p suppress the xenograft tumor growth, we transfected the pRNA-H1.1/Adeno vectors containing the complementary nucleotide sequence of miR-362-5p (anti-miR-362-5p) or NC into SW780 cell and selected using 300 mg/ml G418. We determined the efficiency of anti-miR-362-5p by detecting the expression of CYLD (**Supplementary Figures 1E, H**). The transfected cells were injected into nude mice and the growth of the xenograft tumor was measured. The mice were killed on day 25 after injection, the tumors were collected and displayed (**Figure 5A**), the results showed that the tumor sizes in anti-miR-362-5p transfected cell injected mice were clearly smaller than that in control mice. The tumor volume and weight of the anti-miR-362-5p injected mice were significantly decreased compared to NC injected mice (**Figures 5C, D**). The qRT-PCR results confirmed that the miR-362-5p expression was downregulated in anti-miR-362-5p xenograft tumor tissues compared to control xenograft tumor tissues (**Figure 5B**). The results from immunohistochemistry showed that downregulation of miR-362-5p inhibited the expression of Ki-67, a biomarker of cell proliferation (**Figure 5E**). Moreover, downregulation of miR-362-5p promoted the protein levels of QKI, MacroH2A1.1, PARP-1, p27 in tumor tissues, while reduced the protein levels of Cyclin D, MacroH2A1.2, c-Fos, and E2F1 (**Figure 5F**). The results indicated that downregulation of miR-362-5p suppressed the growth of bladder cancer tumors *in vivo*.

### The Effect of MiR-362-5p on Bladder Cancer Cell Proliferation Could be Regulated by MBNL1-AS1

**Figure 6:**
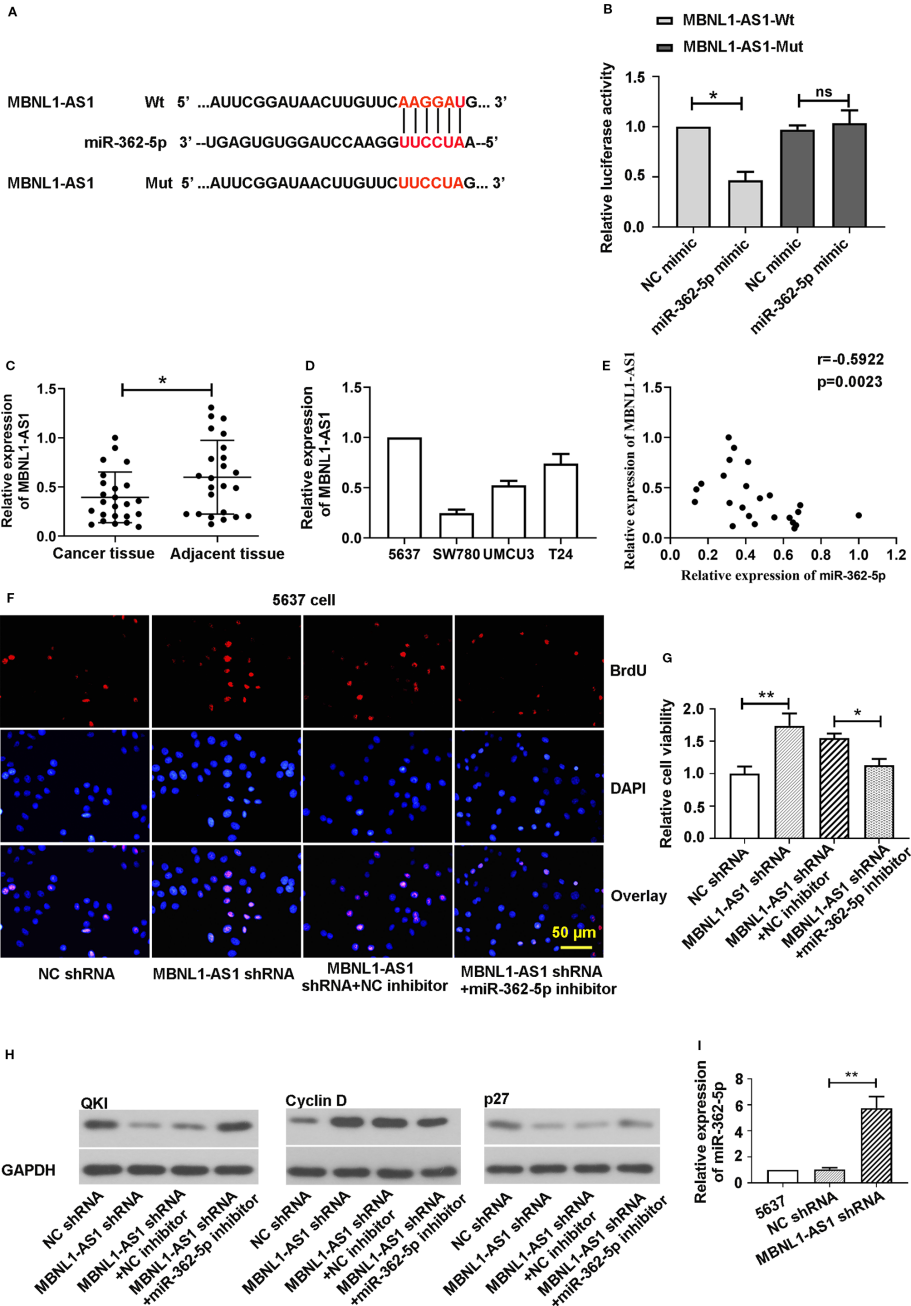
MBNL1-AS1 acts as a sponge for miR-362-5p. **(A)** The predicted binding sites within miR-362-5p and wild-type of MBNL1-AS1 (MBNL1-AS1-Wt) and the mutant sequence of MBNL1-AS1 (MBNL1-AS1-Mut). **(B)** The pmirGLO luciferase vector was inserted with fragments of MBNL1-AS1-Wt or MBNL1-AS1-Mut. Then the constructed pmirGLO luciferase vectors (1 μg) were co-transfected with miR-362-5p mimic (50 pmol) or NC mimic. Luciferase reporter assay was performed after for 48 h transfection. *p < 0.05 *vs*. controls. **(C)** The expression levels of MBNL1-AS1 in bladder cancer tissues (N=24) and adjacent tissues (N=24) were analyzed by qRT-PCR. **p < 0.01 *vs*. adjacent tissues. **(D)** The relative expression levels of MBNL1-AS1 in bladder cancer cell lines 5637, SW780, UMUC3, and T24 were analyzed by qRT-PCR. **(E)** Spearman correlation coefficient analysis was used to evaluate associations between miR-362-5p and MBNL1-AS1 verifed by qRT-PCR (r = −0.5922, p = 0.0023). **(F)** The 5637 cells were co-transfected MBNL1-AS1 shRNA/NC shRNA (1 μg) and miR-362-5p inhibitor/NC inhibitor (50 pmol) for 48 h. Cell proliferation was detected by stainning BrdU in immunofluorescence assay (bar=50 μm). **(G)** The cell viability of transfected cells was measured by MTT assay at 48 h. The cell viability was displayed as fold of NC shRNA. **(H)** The protein levels of QKI, Cyclin D, and p27 was measured by western blot. GAPDH was used as an internal control in western blot. **(I)** The relative expression levels of miR-362-5p in MBNL1-AS1 shRNA or NC shRNA transfected 5637 cells were analyzed by qRT-PCR. The relative expression was displayed as fold of 5637 cells. **p < 0.01 and *p < 0.05 *vs*. corresponding controls.

In order to find the possible target lncRNA that mediates the functions of miR-362-5p in bladder cancer cells, we predicted and verified the specific binding between miR-362-5p and MBNL1-AS1. We the predicted binding sites of miR-362-5p and wild type of lncRNA MBNL1-AS1 (MBNL1-AS1-Wt) and sequence the lncRNA MBNL1-AS1 mutant (MBNL1-AS1-Mut) in red (**Figure 6A**). Dual luciferase reporter assay showed that miR-362-5p mimic could reduce the luciferase activity of MBNL1-AS1-Wt transfected cells but not MBNL1-AS1-Mut transfected cells (**Figure 6B**). Next, we measured the expression of MBNL1-AS1 in bladder cancer tissue and cell lines (**Figures 6C, D**). The results showed the miR-362-5p was negatively correlated with MBNL1-AS1 in bladder cancer, which was verified by the Spearman analysis (r = −0.5922, P = 0.0023) (**Figure 6E**).

For further study, we co-transfected the MBNL1-AS1 shRNA/NC shRNA and miR-362-5p inhibitor/NC inhibitor into 5637 cells. In order to ensure that MBNL1-AS1 shRNA we designed was specific targeting MBNL1-AS1, we used another MBNL1-AS1 shRNA-2 targeting MBNL1-AS1. The results showed that MBNL1-AS1 shRNA and MBNL1-AS1 shRNA-2 both could downregulate MBNL1-AS1 expression (**Supplementary Figure 3A**). Then we found that downregulation of MBNL1-AS1 promoted cell proliferation and cell viability, and silence of miR-362-5p could abate the increased proliferation and viability in the immunofluorescence and MTT assay (**Figures 6F, G**, **Supplementary Figure 3B**). We measured the cell viability using MBNL1-AS1 shRNA-2 and the results showed miR-362-5p inhibitor also attenuated the increased cell viability after transfecting MBNL1-AS1 shRNA-2 (**Supplementary Figure 3C**). Additionally, downregulation of miR-362-5p attenuated the decrease of QKI and p27 protein levels and the increase of Cyclin D expression that caused by knockdown of MBNL1-AS1 (**Figure 6H**).

We also found the expression of miR-362-5p was significantly increased in MBNL1-AS1 shRNA transfected 5637 cells (**Figure 6I**). And upregulation of MBNL1-AS1 decreased the expression of miR-362-5p and increased the expression of QKI (**Supplementary Figures 3D–G**). Moreover, upregulation of miR-362-5p also could decrease the expression of MBNL1-AS1 (**Supplementary Figure 3H**). The results indicated that MBNL1-AS1 mediates the miR-362-5p expression, leading to regulate the proliferation of bladder cancer cells.

## Discussion

One third of all patients may undergo the recurrence of bladder cancer and lead to dramatically decrease the 5-year overall survival. Studies found that the abilities of the proliferation and invasion of bladder cancer cells are involved in the bladder cancer recurrence (Shen et al., 2014; Li et al., 2016; Xiong et al., 2017). It is well known that the proliferation of bladder cancer cells and the growth of bladder tumor are mainly mediated through phosphatidylinositol 3-kinase (PI3K)/AKT pathway (Sun et al., 2018; Cheng et al., 2019). Moreover, a large number of ncRNAs including miRNAs and lncRNAs have been demonstrated to play important roles in bladder cancer progression (Martens-Uzunova et al., 2014; Blanca et al., 2017). In this study, firstly, we found that the expression of miR-362-5p was upregulated in bladder cancer tissues compared with the matched adjacent tissues from the clinical patients. Therefore, we tried to uncover the role of miR-362-5p in bladder cancer and underlying regulatory mechanism. To meet the goal, we carried out the loss- and gain-functions experiments to determine the effect of miR-362-5p on the proliferation of bladder cancer cells *in vitro* and the tumor growth in a mouse model *in vivo*.

MiR-362-5p functions as a critical factor in mediating the malignant behaviors of the tumor cells by regulating the expression of some downstream genes. Studies found that miR-362-5p reduces the expression of tumor suppressor protein CYLD, which leads to activate the NF-*k*B pathway to promote the proliferation, migration, and invasion of hepatocellular carcinoma cells and human breast cancer cells (Ni et al., 2015; Ni et al., 2016). In addition, miR-362-5p directly binds and negatively regulates the expression of Semaphorin 3A (Sema3A) in non-small cell lung cancer (NSCLC) to enhance the cell invasion and migration *in vitro* and tumor formation *in vivo* (Luo et al., 2018). However, miR-362-5p also exhibits a tumor suppressive role in neuroblastoma by inhibiting cell proliferation and migration through PI3K-C2b (Wu et al., 2015). The biological functions of miR-362-5p in bladder cancer are unknown. Our results showed that upregulation of miR-362-5p promoted the proliferation and G1/S transition in cell cycle, while downregulation of miR-362-5p displayed the opposite tendency. The miR-362-5p directly targeted QKI and knockdown of QKI could abate the decreased proliferation and cell cycle progression that induced by miR-362-5p inhibitor. Additionally, we found that the expression of QKI was decreased in bladder cancer tissues. Moreover, the sizes of xenograft tumor formed by injecting cancer cells to nude mice were remarkably decreased in anti-miR-362-5p transfected cells compared to NC transfected cells *in vivo*. Mechanically, silence of miR-362-5p negatively regulated the expression levels of QKI and cell proliferation-related gene proteins MacroH2A1.1, PARP-1, and p27, and positively regulated the expression levels of MacroH2A1.2, Cyclin D, and c-Fos, which could be reversed by knocking down QKI.

The biological functions of QKI have been extensively studied in multiple cancer types by regulating the expression and activity of numbers of functional proteins (Darbelli and Richard, 2016). Leonid Novikov et al. found that QKI is a key factor to mediated alternative splicing of the Histone Variant MacroH2A1 (spliced forms, macroH2A1.1 and macroH2A1.2) to affect the proliferation of cancer cells. Briefly, they claimed that QKI regulates the alternative splicing of macroH2A1 pre-mRNA to produce more macroH2A1.1, which can inhibit cell proliferation by upregulating the expression of PARP-1. Additionally, the expression levels of QKI and macroH2A1.1 were decreased in bladder cancer tissues (Sporn et al., 2009; Novikov et al., 2011). It also reported that QKI-6 inhibits bladder cancer cell proliferation through downregulating E2F3 and NF‐κB pathway (Shi et al., 2019). Yang et al. stated that QKI negatively regulates the expression levels of Cyclin D and c-Fos and positively regulates p27 expression to block the cell cycle progress. Moreover, the expression of QKI can be increased by E2F1, resulting in suppressing the E2F1 activity to delay S-phase entry in cell cycle progression (Yang et al., 2011). Their findings explained, in our result of **Figure 4D**, why knockdown of QKI did not dramatically affect E2F1 expression. Conclusively, the QKI plays an inhibitory role in cell proliferation. Our results indicated that miR-362-5p could promote cell proliferation of bladder cancer cells through regulating QKI.

Bioinformatics analysis and luciferase assay predicted and confirmed that MBNL1-AS1 directly bond with miR-362-5p. Additionally, we found the expression of MBNL1-AS1 was decreased in bladder cancer tissues. The study of the biological function of MBNL1-AS1 is limited. Li et al. found that downregulation of MBNL1-AS1 could enhance the proliferation and suppress the apoptosis of skeletal muscle cells though activating cGMP-PKG signaling pathway, which leads to protect sevoflurane-pretreated mice against I/R injury after total knee arthroplasty (Li et al., 2018). Additionally, the expression of MBNL1-AS1 is decreased in NSCLC tissues compared to adjacent tissues and upregulation of MBNL1-AS1 could inhibit the proliferation of NSCLC cancer stem cell *in vitro* and xenograft tumor formation *in vivo via* miR-301b-3p/transforming growth factor β receptor 2 (TGFBR2) axis (Li et al., 2019b). We verified that overexpression of MBNL1-AS1 could downregulate miR-362-5p level and increase the QKI protein level, and knockdown of MBNL1-AS1 showed the opposite expression trends of miR-362-5p and QKI. Moreover, upregulation of miR-362-5p inhibited the expression level of MBNL1-AS1. The results indicated MBNL1-AS1 functions as ceRNA (sponge) of miR-362-5p by competitively binding to miR-362-5p with mRNA of QKI, leading to mediate the miR-362-5p function. Downregulation of MBNL1-AS1 attenuated the changes of cell proliferation and protein expressions through regulating miR-362-5p. There is a limitation in our study was that we detected the miR-362-5p expression level only using qRT-PCR. That means firstly we need do reverse transcription to obtain cDNA and could not directly detect the microRNA level. However, qRT-PCR still is the most widely used method because qRT-PCR can carry out high-throughput detection with high sensitivity and specificity and it is easy to operate. Therefore, we chose qRT-PCR for miR-362-5p expression detection. Moreover, 24 pairs of clinical samples might be limited for analyzing the correlation between miR-362-5p and clinicopathologic characteristics, and we will keep collecting the samples for further study.

## Conclusions

In conclusion, miR-362-5p exhibits an oncogenic role in bladder cancer by promoting cell proliferation and downregulating miR-362-5p inhibits xenograft tumor growth *in vivo* through QKI. MBNL1-AS1 might function as a (ceRNA) sponge to mediate the miR-362-5p expression and function in bladder cancer cells. To the best of our knowledge, this is the first report of the functions of miR-362-5p in bladder cancer and the potential regulatory mechanism, which might provide a potential therapeutic target in bladder cancer treatment.

## Data Availability Statement

The raw data supporting the conclusions of this article will be made available by the authors, without undue reservation, to any qualified researcher.

## Ethics Statement

The studies involving human participants were reviewed and approved by the Ethics Committee of The First Affiliated Hospital of Zhengzhou University. The patients/participants provided their written informed consent to participate in this study. The animal study was reviewed and approved by Ethics Committee of The First Affiliated Hospital of Zhengzhou University.

## Author Contributions

XW and DS contributed conception and design of the study. QW, XW, BW, and XY organized the database. YY and CY performed the statistical analysis. XW and ZF wrote the first draft of the manuscript. LS, XF, JT, and YG wrote sections of the manuscript. All authors contributed to manuscript revision, read and approved the submitted version.

## Funding

This study was supported by a grant from the Joint Funds of the National Natural Science Foundation of China (No. U1904162).

## Conflict of Interest

The authors declare that the research was conducted in the absence of any commercial or financial relationships that could be construed as a potential conflict of interest.

## Supplementary Material

Supplementary Figure 1
**(A)** The 5637 and T24 cells were transfected with miR-362-5p mimic (100 pmol) or NC mimic for 48 h, and the expression levels of miR-362-5p were measured by RT-PCR. **(B)** The SW780 and UMUC3 cells were transfected with miR-362-5p inhibitor (100 pmol) or NC inhibitor for 48 h, and the expression levels of miR-362-5p were measured by RT-PCR. **(C, F)** The 5637 cells were transfected with miR-362-5p mimic (100 pmol) or NC mimic for 48 h, and the mRNA and protein levels of CYLD were measured by RT-PCR and western blot. **(D, G)** The SW780 cells were transfected with miR-362-5p inhibitor (100 pmol) or NC inhibitor for 48 h, and the mRNA and protein levels of CYLD were measured by RT-PCR and western blot. **(E, H)** A hairpin sequence containing the 100% complementary nucleotide sequence of miR-362-5p was constructed into pRNA-H1.1/Adeno vector. The SW780 cells were transfected with vector containing anti-miR-362-5p (2 μg) or miR-NC for 48 h, and the mRNA and protein levels of CYLD were measured by RT-PCR and western blot. The expression was displayed as fold of 5637 or SW780 cells. **(I)** The 5637 cells were transfected with miR-362-5p mimic (100 pmol) or NC mimic; the SW780 cells were transfected with miR-362-5p inhibitor (100 pmol) or NC inhibitor. After 48 h, the cell proliferation was examined by staining with BrdU in immunofluorescence assay and the percentage of BrdU cells was measured. The percentage of BrdU cells was displayed as fold of 5637 or SW780 cells. **(J, K)** The T24 cells were transfected with miR-362-5p mimic (100 pmol) or NC mimic; the UMUC3 cells were transfected with miR-362-5p inhibitor (100 pmol) or NC inhibitor. After 48 h, the cell proliferation was measured by MTT assay. The cell proliferation was displayed as fold of T24 or UMUC3 cells at 0 h. **p < 0.01 and *p < 0.05 *vs*. corresponding controls.

Supplementary Figure 2
**(A–D)** The 5637 cells and SW780 cells were transfected with QKI siRNA or NC siRNA for 48 h, and the mRNA and protein levels of QKI were measured by RT-PCR and western blot. GAPDH was used as an internal control in western blot. The expression was displayed as fold of 5637 or SW780. **(E, F)** The cell proliferation of QKI siRNA/NC siRNA transfected cells was measured by MTT assay. The cell proliferation was displayed as fold of 5637 or SW780 cells at 0 h. **(G)** The SW780 cells were co-transfected with miR-362-5p inhibitor/NC inhibitor (50 pmol) and QKI siRNA/NC siRNA (50 pmol) for 48 h. Then the cell proliferation was determined by staining BrdU and the percentage of BrdU cells was measured. The percentage of BrdU cells was displayed as fold of NC inhibitor. **p < 0.01 and *p < 0.05 *vs*. corresponding controls.

Supplementary Figure 3
**(A)** Two shRNAs (shRNA, shRNA-2) against MBNL1-AS1 or a negative control shRNA (NC shRNA) were inserted into PRNAH1.1 vector. The 5637 cells were transfected with 2 μg of MBNL1-AS1, shRNA MBNL1-AS1 shRNA-2, or NC shRNA for 48 h. The relative expression of MBNL1-AS1 was analyzed by qRT-PCR. The expression was displayed as fold of 5637 cells. **(B)** The 5637 cells were co-transfected MBNL1-AS1 shRNA/NC shRNA (1 μg) and miR-362-5p inhibitor/NC inhibitor (50 pmol) for 48 h. Cell proliferation was detected by staining BrdU and the percentage of BrdU cells was measured. The percentage of BrdU cells was displayed as fold of NC shRNA. **(C)** The 5637 cells were co-transfected with 1 μg of MBNL1-AS1 shRNA-2/NC shRNA with 50 pmol of miR-362-5p inhibitor/NC inhibitor for 48 h. The cell viability of transfected cells was measured by MTT assay. The cell viability was displayed as fold of NC shRNA. **(D–F)** The adenoviral vectors expressing MBNL1-AS1 (Ad-MBNL1-AS1) or NC (Ad-NC) were constructed and infected the SW780 cells for 24 h, the cells were cultured in complete medium. The relative expression levels of MBNL1-AS1, miR-362-5p, and QKI were analyzed by qRT-PCR after 48 h. The expression was displayed as fold of SW780 cells. **(G)** The protein level of QKI was analyzed by western blot. GAPDH was used as an internal control in western blot. **(H)** The 5637 cells were transfected with miR-362-5p mimic (100 pmol) or NC mimic for 48 h, and the relative expression level of MBNL1-AS1 was measured by RT-PCR. The expression was displayed as fold of 5637 cells. **p < 0.01 and *p < 0.05 *vs*. corresponding controls.

## Abbreviations

QKI, quaking; NMIBCs, non-muscle-invasive bladder cancers; MTT, 3-(4,5-dimethylthiazol-2-yl)-2,5-diphenyl tetrazolium; qRT-PCR, quantitative real-time PCR; HRP, horseradish peroxidase; CYLD, cylindromatosis; NSCLC, non-small cell lung cancer; PI3K, phosphatidylinositol 3-kinase; PARP-1, poly (ADP-ribose) polymerase 1; TGFBR2, transforming growth factor β receptor 2.
