# Supplementary figures and images for: MiR-362-5p, Which Is Regulated by Long Non-Coding RNA MBNL1-AS1, Promotes the Cell Proliferation and Tumor Growth of Bladder Cancer by Targeting QKI
Source: Front Pharmacol. 2020 Mar 3;11:164. doi: 10.3389/fphar.2020.00164 (PMC7063466; doi:10.3389/fphar.2020.00164)

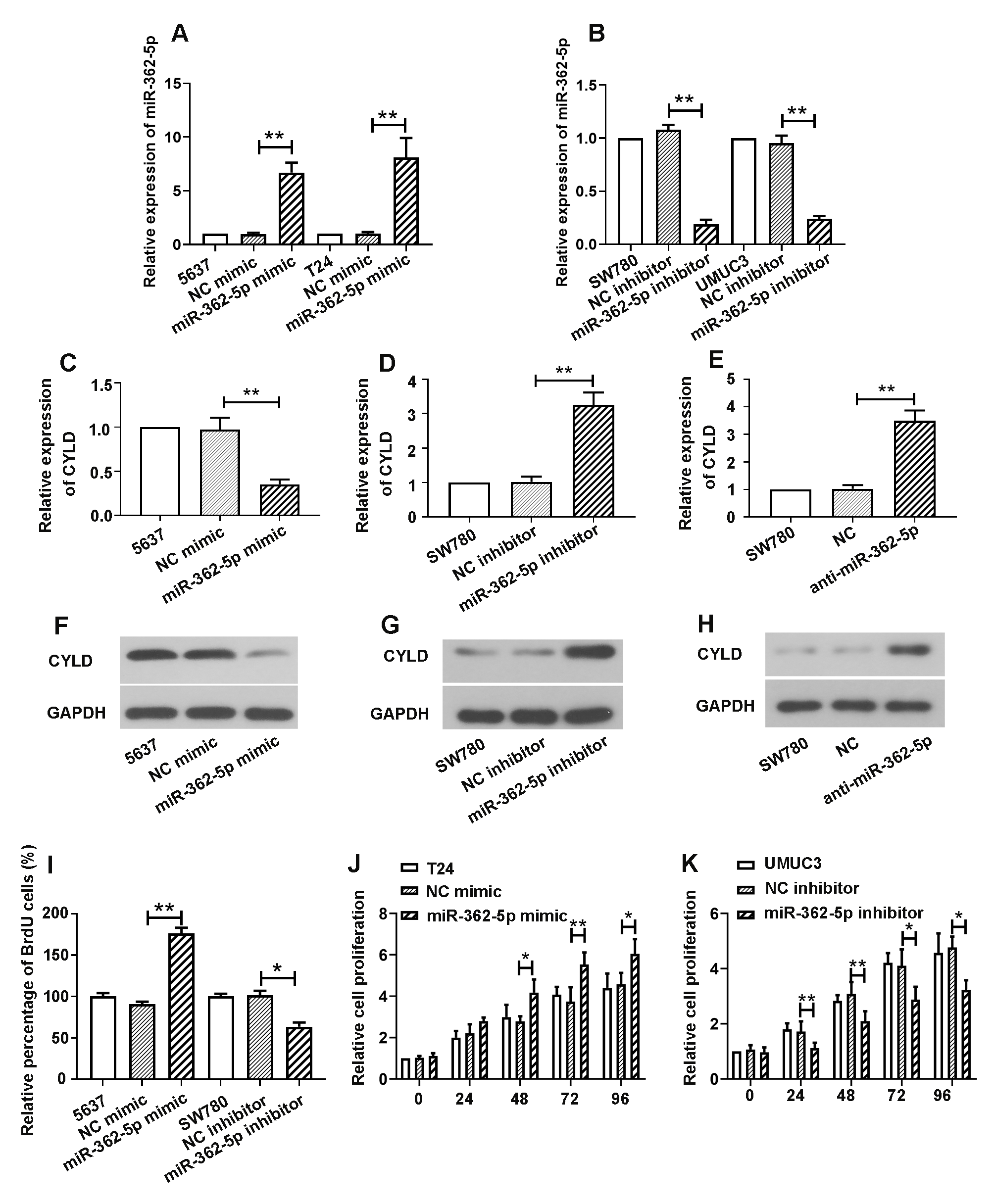

Supplement: Supplementary Figure 1 — (A) The 5637 and T24 cells were transfected with miR-362-5p mimic (100 pmol) or NC mimic for 48 h, and the expression levels of miR-362-5p were measured by RT-PCR. (B) The SW780 and UMUC3 cells were transfected with miR-362-5p inhibitor (100 pmol) or NC inhibitor for 48 h, and the expression levels of miR-362-5p were measured by RT-PCR. (C, F) The 5637 cells were transfected with miR-362-5p mimic (100 pmol) or NC mimic for 48 h, and the mRNA and protein levels of CYLD were measured by RT-PCR and western blot. (D, G) The SW780 cells were transfected with miR-362-5p inhibitor (100 pmol) or NC inhibitor for 48 h, and the mRNA and protein levels of CYLD were measured by RT-PCR and western blot. (E, H) A hairpin sequence containing the 100% complementary nucleotide sequence of miR-362-5p was constructed into pRNA-H1.1/Adeno vector. The SW780 cells were transfected with vector containing anti-miR-362-5p (2 μg) or miR-NC for 48 h, and the mRNA and protein levels of CYLD were measured by RT-PCR and western blot. The expression was displayed as fold of 5637 or SW780 cells. (I) The 5637 cells were transfected with miR-362-5p mimic (100 pmol) or NC mimic; the SW780 cells were transfected with miR-362-5p inhibitor (100 pmol) or NC inhibitor. After 48 h, the cell proliferation was examined by staining with BrdU in immunofluorescence assay and the percentage of BrdU cells was measured. The percentage of BrdU cells was displayed as fold of 5637 or SW780 cells. (J, K) The T24 cells were transfected with miR-362-5p mimic (100 pmol) or NC mimic; the UMUC3 cells were transfected with miR-362-5p inhibitor (100 pmol) or NC inhibitor. After 48 h, the cell proliferation was measured by MTT assay. The cell proliferation was displayed as fold of T24 or UMUC3 cells at 0 h. **p < 0.01 and *p < 0.05 vs. corresponding controls. [file Image_1.tif]

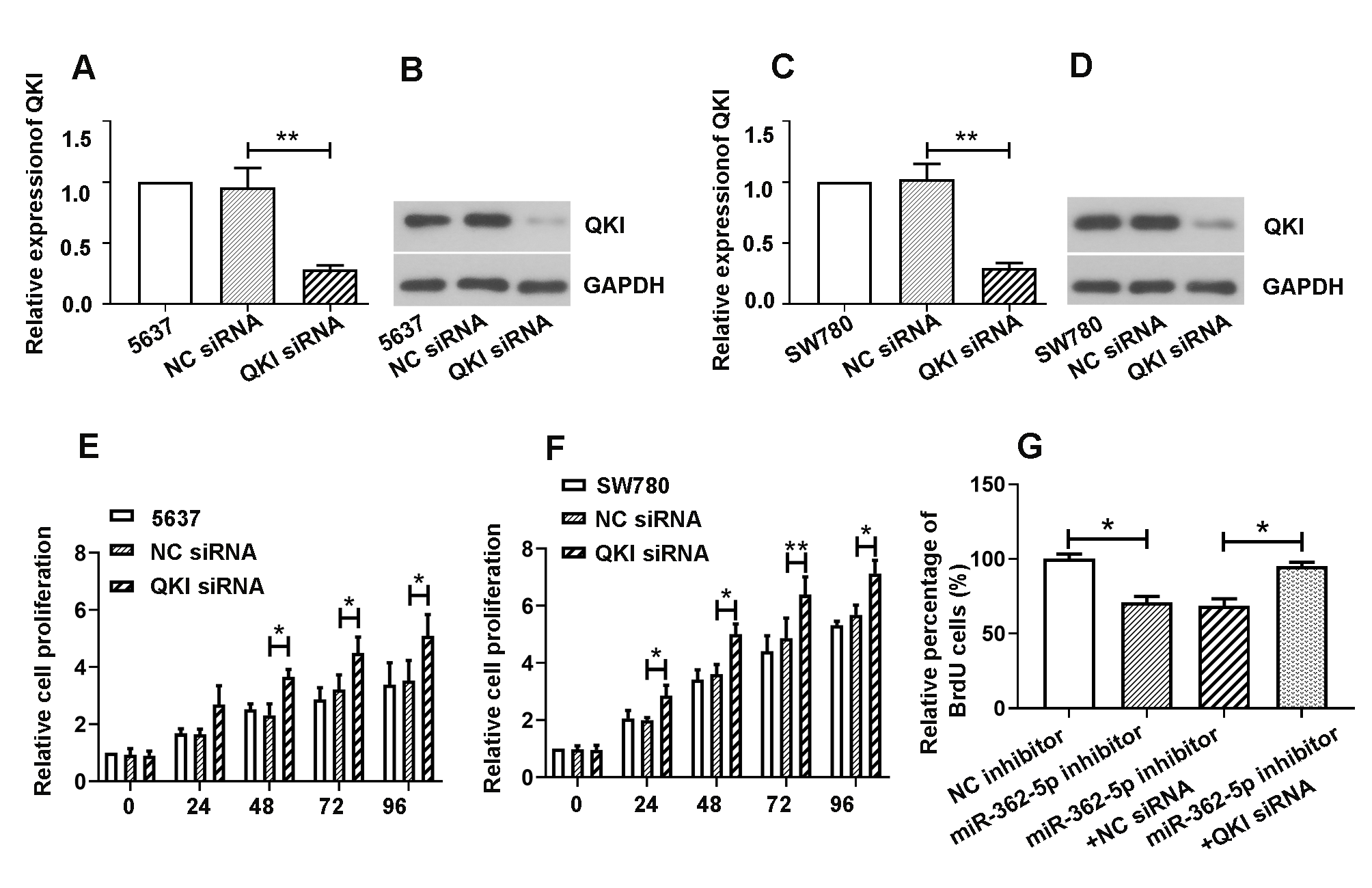

Supplement: Supplementary Figure 2 — (A–D) The 5637 cells and SW780 cells were transfected with QKI siRNA or NC siRNA for 48 h, and the mRNA and protein levels of QKI were measured by RT-PCR and western blot. GAPDH was used as an internal control in western blot. The expression was displayed as fold of 5637 or SW780. (E, F) The cell proliferation of QKI siRNA/NC siRNA transfected cells was measured by MTT assay. The cell proliferation was displayed as fold of 5637 or SW780 cells at 0 h. (G) The SW780 cells were co-transfected with miR-362-5p inhibitor/NC inhibitor (50 pmol) and QKI siRNA/NC siRNA (50 pmol) for 48 h. Then the cell proliferation was determined by staining BrdU and the percentage of BrdU cells was measured. The percentage of BrdU cells was displayed as fold of NC inhibitor. **p < 0.01 and *p < 0.05 vs. corresponding controls. [file Image_2.tif]

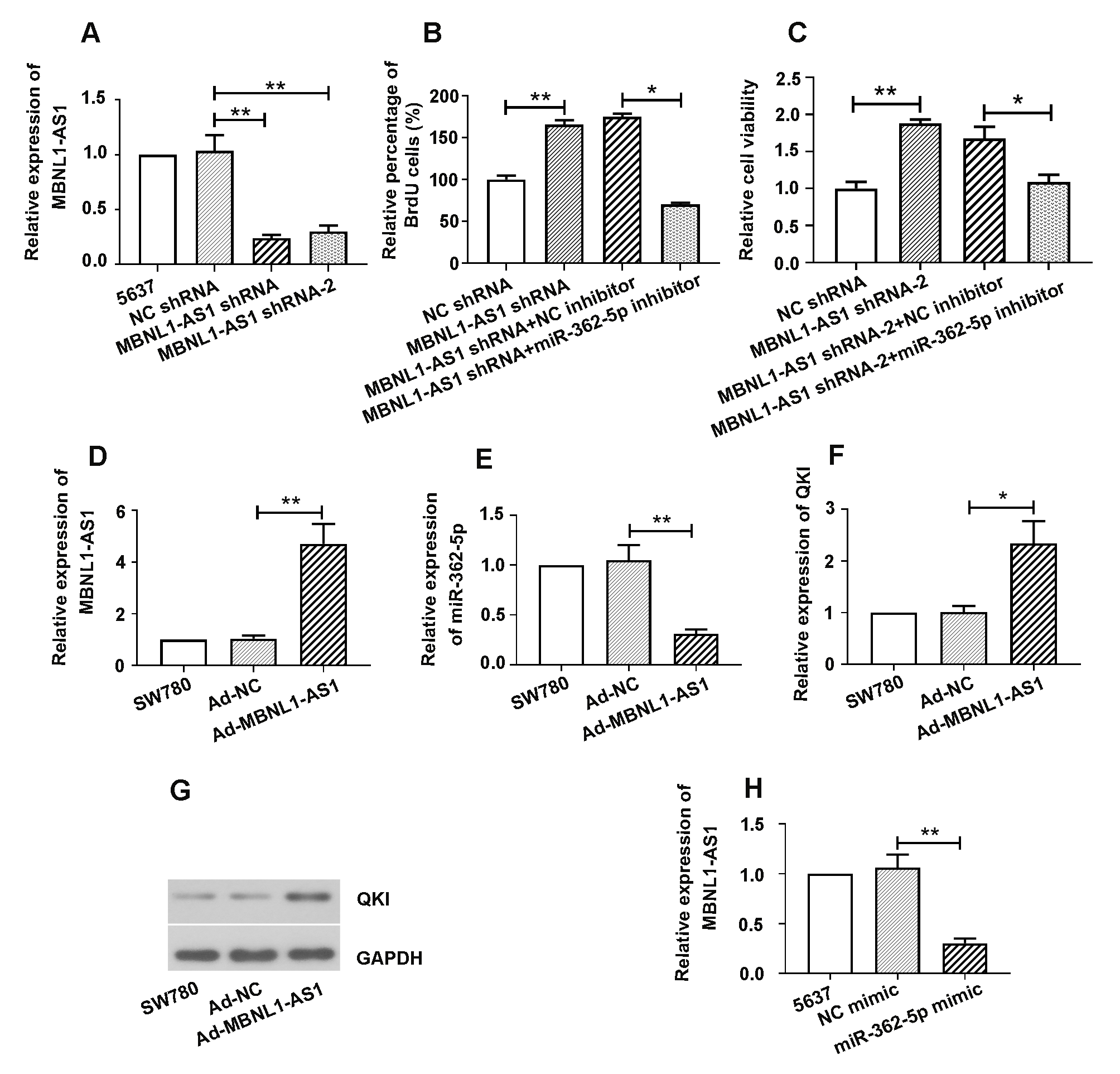

Supplement: Supplementary Figure 3 — (A) Two shRNAs (shRNA, shRNA-2) against MBNL1-AS1 or a negative control shRNA (NC shRNA) were inserted into PRNAH1.1 vector. The 5637 cells were transfected with 2 μg of MBNL1-AS1, shRNA MBNL1-AS1 shRNA-2, or NC shRNA for 48 h. The relative expression of MBNL1-AS1 was analyzed by qRT-PCR. The expression was displayed as fold of 5637 cells. (B) The 5637 cells were co-transfected MBNL1-AS1 shRNA/NC shRNA (1 μg) and miR-362-5p inhibitor/NC inhibitor (50 pmol) for 48 h. Cell proliferation was detected by staining BrdU and the percentage of BrdU cells was measured. The percentage of BrdU cells was displayed as fold of NC shRNA. (C) The 5637 cells were co-transfected with 1 μg of MBNL1-AS1 shRNA-2/NC shRNA with 50 pmol of miR-362-5p inhibitor/NC inhibitor for 48 h. The cell viability of transfected cells was measured by MTT assay. The cell viability was displayed as fold of NC shRNA. (D–F) The adenoviral vectors expressing MBNL1-AS1 (Ad-MBNL1-AS1) or NC (Ad-NC) were constructed and infected the SW780 cells for 24 h, the cells were cultured in complete medium. The relative expression levels of MBNL1-AS1, miR-362-5p, and QKI were analyzed by qRT-PCR after 48 h. The expression was displayed as fold of SW780 cells. (G) The protein level of QKI was analyzed by western blot. GAPDH was used as an internal control in western blot. (H) The 5637 cells were transfected with miR-362-5p mimic (100 pmol) or NC mimic for 48 h, and the relative expression level of MBNL1-AS1 was measured by RT-PCR. The expression was displayed as fold of 5637 cells. **p < 0.01 and *p < 0.05 vs. corresponding controls. [file Image_3.tif]
